# Use of botulinum toxin injections to treat peripheral stimulator induced facial muscle twitching: a case report

**DOI:** 10.1186/s40064-015-1473-x

**Published:** 2015-11-04

**Authors:** Terrence L. Trentman, Jillian A. Maloney, Christopher S. Wie, Alanna M. Rebecca, David M. Rosenfeld

**Affiliations:** Department of Anesthesiology and Pain Medicine, Mayo Clinic Arizona, 5777 E Mayo Blvd, Phoenix, AZ 85054 USA; Valley Pain Consultants, Phoenix, AZ USA; Department of Plastics and Reconstructive Surgery, Mayo Clinic Arizona, 5777 E Mayo Blvd, Phoenix, AZ 85054 USA

## Abstract

**Background:**

Facial pain can be a management challenge. Peripheral nerve/field stimulation may be an effective option for refractory cases, but direct muscle stimulation with facial twitching may result. Botulinum toxin injections have been used for blepharospasm and may be effective when facial stimulation results in unacceptable facial muscle twitching due to peripheral stimulation.

**Case presentation:**

A 53-year old female suffered with chronic, refractory facial pain and migraines. Her facial pain began after a root canal of a left upper molar. She was trialed and then permanently implanted with a 1 × 8 sub-compact percutaneous stimulator lead, resulting in improved pain control and reduced medication use. However, she experienced blepharospasm whenever the amplitude was above 2.75 A. Therefore, she was treated with botulinum toxin injections into her bilateral cheek, face, temple and occiput. This treatment provided excellent relief of the facial spasms, allowing her to use her stimulator at high amplitudes, and thereby maximizing her pain relief. She received two subsequent treatments of botulinum toxin injections at 5-month intervals with similar results.

**Conclusion:**

Peripheral nerve/field stimulation is being used for headaches and facial pain. An undesirable side effect of this emerging therapy is direct muscle stimulation. Botulinum toxin injections may be an effective treatment modality when stimulation techniques provide pain relief but also causes muscle twitching.

## Background

Peripheral nerve or field stimulation is used for a variety of chronic pain conditions, including headache and facial pain (Dodick et al. 2015; Sweet et al. 2015; Slavin et al. 2006; Yakovlev and Resch 2010). A number of complications and side effects have been reported after peripheral nerve and field stimulation of the head and face, including painful stimulation, lead erosion, lead migration, and direct muscle stimulation (Schwedt et al. 2007; Sharan et al. 2015; Trentman et al. 2008; Zach et al. 2014).

Inserting effective peripheral leads in the occipital, supraorbital or infraorbital regions, while simultaneously avoiding uncomfortable direct muscle stimulation, can be challenging. These areas have dense musculature which is needed for head movement and facial expression. Minimal subcutaneous fat can complicate the challenge of inserting leads in an anatomic plane that provides clinical benefit without muscle twitching. Although intraoperative checks are typically carried out in an effort to ensure no muscle response to peripheral stimulation, occasionally patients do experience muscle twitching after occipital or facial stimulator placement, particularly when higher stimulation amplitudes are used.

This case report describes successful use of botulinum toxin injections to treat infraorbital twitching of the orbicularis oculi and zygomaticus major muscles after a permanent peripheral stimulator lead was placed for chronic facial pain. The use of botulinum toxin in this context may offer relief for patients when reprogramming of stimulation parameters is ineffective and the patient desires continued use of the device.

## Case presentation

A 53-year-old female with a past medical history of chronic odontalgia and facial pain was seen in consultation for consideration of a facial stimulator trial. She also suffered chronic migraine headaches. Her facial pain began approximately 8 years earlier after a root canal of an upper left molar which was reportedly complicated by retention of a metal fragment, trigeminal nerve damage (V2 distribution) and chronic left facial pain. She suffered incapacitating pain refractory to treatment with pregabalin, tramadol, tricyclic antidepressants, clonazepam, carbamazepine, topical lidocaine, methadone, transmucosal fentanyl, sphenopalatine ganglion blocks, transcutaneous electrical nerve stimulation (TENS), acupuncture, psychological approaches to pain, facial muscle steroid injections and radiofrequency ablation of the trigeminal ganglion.

**Fig. 1:**
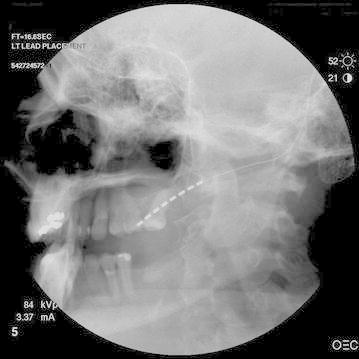
Lateral skull film demonstrating the location of the peripheral stimulation lead in relation to the left upper molars

After a successful percutaneous trial of facial stimulation the patient was taken to the operating room for permanent implant. After induction of general anesthesia, a 14-gauge Tuohy needle was inserted subcutaneously through an incision on the left cheek. Under fluoroscopic guidance, a 1 × 8 sub-compact percutaneous lead (Medtronic, Minneapolis, MN, USA) was inserted over the left maxilla, with care to place the distal 2 contact points next to the upper molars. Several adjustments to the lead were necessary before a position was found where no muscle twitching was observed upon testing, even at high amplitude and low frequency stimulation (e.g. 10 A, 2 Hz), see Fig. 1.

Next, the lead was tunneled from the cheek to a temporal incision, where it was looped and then tunneled via a posterior auricular route to an implantable pulse generator (IPG, RestoreUltra, Medtronic, Minneapolis, MN, USA) in the infraclavicular region. Because of concerns over erosion of bulky commercial anchors, 2-0 silk suture was used to anchor the lead.

On follow-up the patient noted improved pain control and reduced pain medication consumption with her facial stimulator; however, when she increased the amplitude above 2.75, she experienced blepharospasm. After reprogramming failed to remedy this issue she was subsequently treated with freshly prepared botulinum toxin, 100 units/4 ml. This was injected into her bilateral cheeks and face, temple and occiput. The facial injections were based on observed muscle stimulation when the patient adjusted the amplitude of the device. Each facial injection was 0.05 ml which is 1.25 units of botulinum toxin. The temple and occipital regions were treated because of the patient’s history of migraine headaches.

This treatment provided excellent relief of the facial spasms, allowing her to use her stimulator at high amplitudes, thereby maximizing her pain relief. She received two subsequent treatments of botulinum toxin injections at 5-month intervals with similar results. Her facial twitching began to return after about 3 months, but she chose to wait 2 additional months before returning to clinic.

Unfortunately, the following year the patient developed an infection near the post-auricular lead, and she had previous unrelated methicillin resistant staff aureus (MRSA) infections on the contralateral face. Due to the risk of recurrent MRSA infection combined with a planned instrumented spinal fusion for severe adult onset scoliosis, a decision was made to explant the system to reduce the risk of seeding her spinal hardware. After explantation of her stimulator system, her facial pain returned to baseline. She has been maintained on oral opiates and at last follow-up, she was inquiring about any other pain management options.

## Discussion

This case illustrates a unique potential therapy for direct muscle stimulation associated with a peripheral stimulator: intermittent botulinum toxin injections. Although a number of studies discuss botulinum injections for hemifacial spasm, eyelid spasm, Meige Syndrome (oral facial dystonia) and blepharospasm (Ababneh et al. 2014; Czyz et al. 2013), we are unaware of reports of successful treatment of stimulator related muscle contraction. In the present case, the botulinum toxin allowed efficacious stimulation until the device was removed as a risk reduction measure for other comorbidities.

Botulinum toxin is produced by Clostridium botulinum and there are several commercially available formulations of the toxin for medical use, e.g. OnabotulinumtoxinA and RimabotulinumtoxinB. The toxin prevents the release of acetylcholine at the neuromuscular junction and thereby inhibits muscular contraction. The Food and Drug Administration (FDA) has approved its use for multiple conditions including the treatment of chronic migraine, cervical dystonia, severe primary axillary hyperhidrosis, glabellar rhytides (skin wrinkling), lateral canthal lines (crow’s feet), strabismus, blepharospasm, and hemifacial spasm.

Of particular interest in pain management practice is the recent and evolving use of botulinum toxin for chronic migraine, defined as a headache frequency ≥15 days per month for 3 months in the absence of medication overuse. However, chronic migraine must be distinguished from medication overuse headache (Negro et al. 2011). Recent post-marketing data supports the use of OnabotulinumtoxinA for chronic migraine: among 254 adults treated with OnabotulinumtoxinA, the number of headache and migraine days were significantly reduced, and the number of headache free days were increased (Khalil et al. 2014). In the case presented here, the botulinum toxin may have had a synergistic pain relieving effect when combined with the stimulation, considering botulinum toxin’s pain relieving effects as seen in chronic migraine.

Botulinum toxin injection is not without risks and financial considerations. The FDA warns of the risks of injection of botulinum toxin including unexpected muscle weakness, hoarseness, dysarthria, loss of bladder control, respiratory distress, dysphagia, blurred vision, diplopia and ptosis.

Previous studies have discussed complications of head and neck peripheral stimulation (Palmisani et al. 2013). Lead migration is a well-known adverse event, in addition to complications like lead erosion, painful stimulation and infection. Hayek et al. discussed muscle spasms from occipital stimulation (Hayek et al. 2009). They described placing the leads higher at the nuchal line, rather than at the C1 level which had been previously reported (Weiner and Reed 1999). They found that higher lead placement eliminated neck muscle spasm in 5 patients.

In addition to peripheral stimulation for refractory headaches, a recent prospective, open-label study of cervical spinal cord stimulation (CSCS) in 17 chronic migraine subjects was carried out (Arcioni et al. 2015). The authors found that paresthesia free, 10 kHz CSCS provided >30 % reduction in headache days for 50 % of the 14 patients still implanted with a permanent system at 6 months. Among the responders, the average reduction in headache days was 12.9.

Further studies are needed to validate neuromodulation treatments for refractory headaches. An international consensus gave recommendations for further studies of this therapy and suggested that neurostimulation devices should be reserved for refractory patients as part of research or only after controlled studies have shown the treatment to be effective (Martelletti et al. 2013).

## Conclusion

In facial field stimulation, it may not be possible to avoid some degree of muscle stimulation resulting in undesirable twitching or spasm. In our case, every effort was made to identify and avoid muscle movement during implantation. Postoperatively, the patient suffered significant facial muscle spasm related to stimulator use, but this was successfully treated with botulinum toxin. Further studies are needed to confirm botulinum toxin as a reliable treatment for stimulator induced muscle spasm.

## Consent

Written informed consent was obtained from the patient for publication of this Case Report and any accompanying images. A copy of the written consent is available for review by the Editor-in-Chief of this journal.
